# Epidemiological studies on the health impact of air pollution in Japan: their contribution to the improvement of ambient air quality

**DOI:** 10.1265/ehpm.25-00020

**Published:** 2025-04-26

**Authors:** Masayuki Shima

**Affiliations:** Department of Public Health, School of Medicine, Hyogo Medical University, Nishinomiya 663-8501, Japan

**Keywords:** Automobile exhaust, Bronchial asthma, Climate change, Fine particulate matter (PM_2.5_), Nitrogen dioxide, Ozone, Sulfur dioxide

## Abstract

In Japan, during the high economic growth period (1950–1960s), air pollution due to sulfur dioxide (SO_2_) and dust derived from large-scale factories and power plants was apparent in many industrial districts, and it caused serious health problems such as the so-called “Yokkaichi Asthma.” Many epidemiological studies have revealed the relationship between air pollution and respiratory diseases, and have provided scientific evidence for the regulatory control of air pollution. The concentration of SO_2_ has markedly decreased since the 1970s, and its adverse health effects have improved. In contrast, increased automobile traffic has caused considerable traffic-related air pollution, including nitrogen oxides (NOx) and particulate matter (PM). Epidemiological studies in Chiba and Tokyo revealed that the prevalence and incidence of asthma were significantly higher among individuals living in roadside areas than among those living in other areas. Large-scale epidemiological studies conducted in urban districts have revealed an association between traffic-related air pollution and the onset of asthma in schoolchildren and persistence of asthmatic symptoms in preschool children. Thereafter, the concentrations of NOx and PM gradually decreased due to the control measures based on the Automobile NOx/PM Law enforced in 2001. Thus, epidemiological studies have contributed to a reduction in air pollution caused by automobile exhaust emissions. Recently, the adverse health effects of ambient fine PM (PM_2.5_) and ozone (O_3_) at ground level have become an international concern. Our epidemiological studies showed that short-term exposure to considerably low concentrations of PM_2.5_ and O_3_ was associated with a decrease in pulmonary function among asthmatic children and increased airway inflammation in healthy adolescents. The effects of exposure to PM_2.5_ during pregnancy and early childhood on children’s development have also been reported. These air pollutants consist of not only emissions from primary sources but also secondary formations in the atmosphere. They are affected by climate change and spread worldwide. Air quality control measures and climate change adaptation and mitigation strategies are synergistic, and will have co-benefits on human health. Therefore, global efforts are required to protect populations from the health risks posed by these air pollutants.

## Graphical Abstract

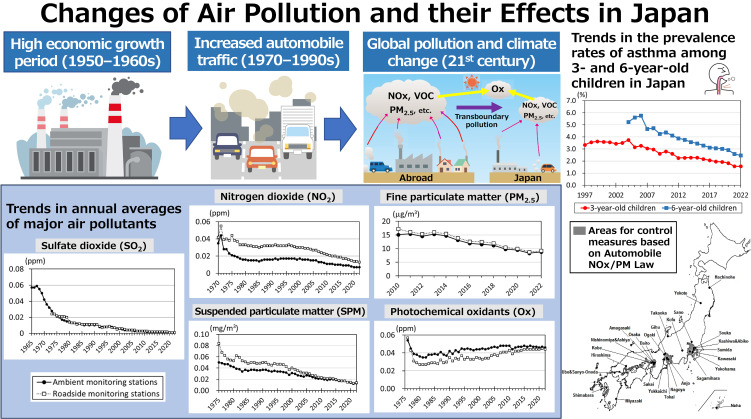

## Background

There are various air pollutants, including particulate matter (PM), carbon monoxide (CO), ozone (O_3_), nitrogen dioxide (NO_2_), and sulfur dioxide (SO_2_), in atmospheric environment [1]. It is well known that air pollutants cause respiratory and cardiovascular diseases in humans. As air pollutants are derived from the use of fossil fuels associated with industrialization and increasing automobile traffic, their impact on human health has been considered previously, mainly in industrial and urban districts in developed countries. In recent years, economic growth has become significant in many emerging countries in Asia, Africa, and elsewhere, and industrialization and motorization have rapidly progressed. Air pollution is a significant global environmental and health concern.

The World Health Organization (WHO) stated that air pollution is one of the greatest environmental risks and affects human health at even lower concentrations than previously understood and updated the Air Quality Guidelines in 2021 [2]. The WHO estimated that ambient air pollution caused 4.2 million premature deaths worldwide in 2019, and that reducing air pollution levels is necessary to protect populations from health risks [3]. This article provides an overview of epidemiological studies on the health effects of air pollution in Japan and their role in the improvement of ambient air quality, focusing on those conducted by the author.

## Air pollution during the high economic growth period (1950–1960s)

Industrialization progresses rapidly in many developed countries during the latter half of the 20^th^ century. Economic development due to industrialization improved the people’s standard of living. However, the expansion of industry has led to the use of vast amounts of resources and energy, generating environmental pollution. In Japan, during the period of high economic growth (1950–1960s) after World War II, heavy and chemical industries developed rapidly. During the period, large quantities of air pollutants, such as SO_2_ and dust, were emitted from factories and power plants in many industrial districts [4], and the serious health problems such as the so-called “Yokkaichi Asthma” were apparent. Many epidemiological studies have investigated the relationship between air pollution and respiratory diseases and provided scientific evidence for the regulatory control of air pollution to protect human health [5, 6]. These findings led to the establishment of *the Basic Law for Environmental Pollution Control* in 1967 and *the Air Pollution Control Law* in 1968. Atmospheric quality standards targeting health problems were established in 1969 and various measures have been implemented to reduce air pollution [4].

**Fig. 1:**
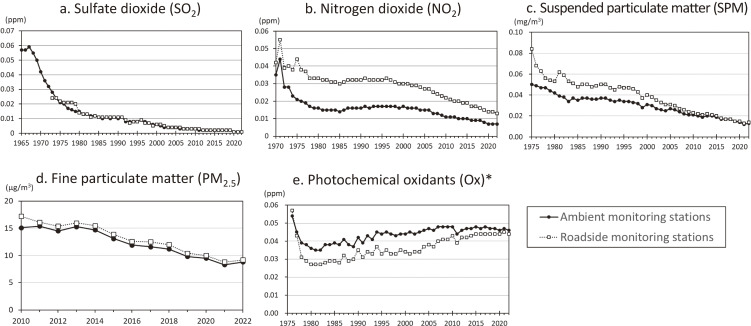
Trends in annual averages of major air pollutants in Japan. *Annual averages of daily maximum 1-hour concentrations during the daytime are shown for photochemical oxidants. Source: Annual report on the continuous monitoring of air pollutants (Ministry of the Environment Government of Japan).

Thereafter, the annual average concentration of atmospheric SO_2_ decreased, following its peak in 1967 (Fig. 1a). An epidemiological study in Yokkaichi reported that mortality due to respiratory diseases in polluted districts decreased in response to improvements in air pollution, suggesting the prevention of adverse health risks due to air pollution [7]. Currently, the concentrations of SO_2_ are very low throughout Japan, except in some districts affected by volcanic eruptions. However, the concentrations of SO_2_ remain high in low- and middle-income countries, and the effects on human health are concerning [8]. Japan’s experience with air pollution control and air quality improvement can be useful for many developing countries facing serious air pollution.

## Health effects of traffic-related air pollution (TRAP)

### (1) Epidemiological studies in Chiba and Tokyo Prefectures

Automobile traffic in Japan has rapidly increased since the 1970s. Motor vehicles emit various pollutants, including PM derived from diesel engines, nitrogen oxides (NOx), polycyclic aromatic hydrocarbons, and volatile organic compounds. Other pollutants, such as the resuspension of road dust, wear of brakes and tires, and abrasion of road surfaces, should also be considered. These are collectively referred to as TRAP [9]. PM and NOx concentrations were considerably higher in areas adjacent to major roadways with heavy traffic in large cities, and their potential health effects have been a major concern. Epidemiological studies were conducted to investigate the effects of TRAP on the health of residents living near major roadways in Chiba Prefecture and the Tokyo Metropolitan area, Japan [10–12].

A cohort study on approximately 5,000 schoolchildren was conducted in 1986 in 10 different communities in Chiba Prefecture [13, 14]. During the study period, the prevalence of asthma was higher among children who lived less than 50 m from trunk roads with heavy traffic than among those in the other areas studied. Additionally, the incidence of asthma during the follow-up period significantly increased among boys living in roadside areas compared with those living in rural areas (odds ratio (OR) = 3.75 [95% confidence interval (CI): 1.00, 14.06]). Among girls, the incidence of asthma also increased (OR = 4.06 [95% CI: 0.91, 18.10]), although the risk was not significant. Additionally, indoor NO_2_ concentrations were measured over 24 h in both winter and summer in the homes of 1,029 children, and the 3-year average of the outdoor NO_2_ concentration was determined for each community. Outdoor NO_2_ concentrations were significantly associated with the incidence of wheezing and asthma (OR = 1.76 [95% CI: 1.04, 3.23] and OR = 2.10 [95% CI: 1.10, 4.75] for a 10-ppb increase, respectively), but no such associations were observed with indoor NO_2_ concentrations (OR = 0.73 [95% CI: 0.45, 1.14] and OR = 0.87 [95% CI: 0.51, 1.43], respectively) [15, 16]. These findings suggest that TRAP may play an important role in the development of wheezing and asthma in children living near major trunk roads with heavy traffic. Outdoor, rather than indoor, NO_2_ concentrations may act as a surrogate for the actual cause of health effects.

In the Tokyo Metropolitan area, respiratory symptoms were investigated in 5,682 adult females in 1987, of whom 733 underwent pulmonary function tests during 8 years from 1987 to 1994. Individuals living in roadside areas with high levels of air pollution showed higher prevalence rates of respiratory symptoms and a larger decrease in forced expiratory volume in 1 s (FEV_1_) than those living in areas with low levels of air pollution [17].

### (2) Study on respiratory diseases and automobile exhaust (SORA project)

The results of studies in Chiba that suggested a relationship between the incidence of asthma and traffic-related exposure have attracted international attention [18], although these studies were conducted in only a limited number of districts. Thus, well-designed, large-scale studies are required to confirm the association between TRAP and the incidence of asthma. Therefore, the Japanese Ministry of the Environment decided to conduct the SORA project. In this project, three large-scale epidemiological studies (a cohort study of schoolchildren, a nested case-control study of preschool children, and a survey of respiratory diseases among adults aged ≥40 years) were conducted in three metropolitan areas [19]. In these studies, the concentrations of elemental carbon (EC) and NOx were estimated for each participant using a high spatial resolution model as an indicator of exposure to emissions from automobile traffic [20].

**Fig. 2:**
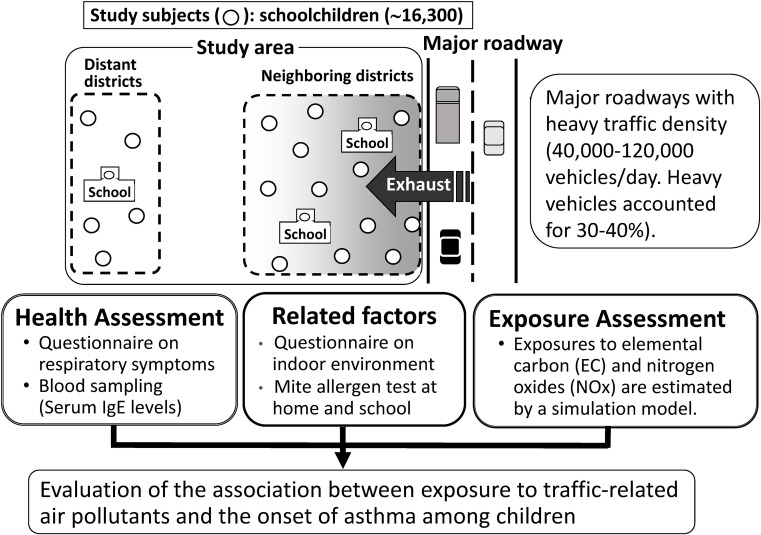
Schematic outline of the cohort study on respiratory diseases and automobile exhaust in schoolchildren

In the cohort study of schoolchildren, 10,069 children aged 6–9 years without asthma were enrolled and followed-up for 4 years (Fig. 2). During the study period, 309 (3.1%) children developed asthma. After adjustment for confounding factors, a positive association between personal exposure to EC in the past 2 years and the incidence of asthma was observed (OR = 1.07 [95% CI: 1.01, 1.14] for a 0.1 µg/m^3^ increase in mean EC concentration). The association between exposure to NOx and the incidence of asthma was not significant (OR = 1.01 [95% CI: 0.99, 1.03] for a 1 ppb increase in mean NOx concentration over the past 2 years) [21]. This suggests an association between TRAP exposure and the onset of asthma in schoolchildren.

In a nested case-control study of preschool children, a baseline survey was conducted with 1.5-year-old children (n = 63,266). In a follow-up survey at 3 years of age (n = 43,343), new-onset asthma (n = 853) and persistent asthma (n = 214) were identified. There was no significant association between the incidence of asthma in children aged 1.5 and 3 years and personal exposure to EC or NOx in the first 1.5 years. However, the persistence of asthmatic symptoms was significantly associated with exposure to NOx (OR = 6.02 [95% CI: 1.51, 23.92] for the comparison between the upper 5th and lower 25th centiles of NOx) [22]. These results support the role of TRAP in the persistence of asthma in preschool children.

In a study of adults over 40 years of age, the analysis of all participants (n = 111,318) showed no association between new-onset asthma within 4 years and personal exposure to EC or NOx. However, in the analysis of only non-smokers (n = 54,568), new-onset of asthma was significantly associated with exposure to EC (OR = 13.9 [95% CI: 1.19, 161.0] for the comparison between the upper 5th and lower 25th centiles of EC) [19].

The SORA project has several limitations. First, the most severe bias of the project was that EC and NOx concentrations decreased over the study period. Therefore, analyses were conducted using discrete-time logistic regression models, expecting little to no bias in the estimated risks [21]. Second, although the exposure assessment improved over previous studies, it remains inadequate for assessing actual personal exposure to TRAP. Third, asthma incidence was evaluated using a questionnaire, not by a clinical diagnosis. However, to support the evaluation of asthma or allergic diseases, the participants’ predisposition to allergy was evaluated by measuring the concentrations of total and specific IgEs against common allergens using blood samples. Despite these limitations, the author believes that the results of these studies provide scientific evidence for reducing the air pollution caused by automobile exhaust and contribute to the improvement in ambient air quality.

### (3) Measures to control automobile exhaust and its public health impacts

In Japan, *the Law Concerning Special Measures for Total Emission Reduction of Nitrogen Oxides (Automobile NOx Law)* was enacted in 1992 to reduce the concentration of NOx derived from automobiles in large cities. In 2001, the law was amended to *the Automobile NOx/PM Law* to include PM in the scope of measures [23]. Thereafter, the concentrations of ambient NO_2_ and suspended PM (SPM) gradually decreased in large cities with heavy traffic (Fig. 1b and c).

The relationship between the decrease in air pollutants and the prevalence of allergic diseases among children was investigated using data from the Environmental Health Surveillance for Air Pollution conducted by the Japanese Ministry of the Environment [24]. This is a large-scale cross-sectional study on respiratory and allergic symptoms among 3- and 6-year-old children in approximately 40 regions throughout Japan, using a questionnaire completed by parents, and has been carried out annually since 1996. The data of 618,973 3-year-old children collected from 28 regions in which the survey was conducted continuously from 1997 to 2009 were analyzed [25].

**Fig. 3:**
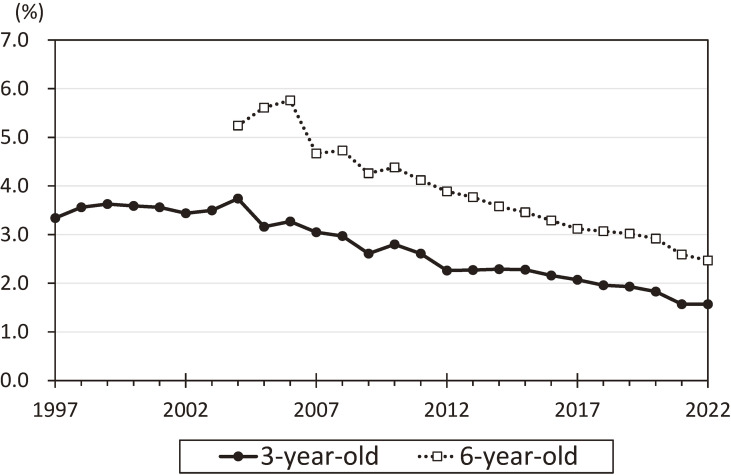
Trends in the prevalence rates of asthma among 3- and 6-year-old children in Japan. The prevalence rates for each year in all the study districts of Japan covered by the Environmental Health Surveillance for Air Pollution are shown after adjusting for a history of allergic diseases in the participants and their parents. Source: Annual Report of Environmental Health Surveillance for Air Pollution (Ministry of the Environment Government of Japan).

Multiple linear regression analysis showed that a reduction in ambient NO_2_ concentration of 1 ppb was significantly associated with a reduction in the prevalence rates of asthma and atopic dermatitis by 0.12% (95% CI: 0.01, 0.23) and 0.39% (95% CI: 0.11, 0.67), respectively. A reduction in SPM by 1 µg/m^3^ was also associated with a reduction in the prevalence of asthma and atopic dermatitis by 0.05% (95% CI: 0.02, 0.08) and 0.14% (95% CI: 0.06, 0.22), respectively. This study suggests the public health benefits of measures to control automobile exhaust emissions improve air quality. These measures contribute to a decrease in the prevalence of asthma and atopic dermatitis in children [25]. The results of the Environmental Health Surveillance for Air Pollution [24] showed that the prevalence rates of asthma among 3- and 6-year-old children in all study districts throughout Japan have decreased since the late 2000s (Fig. 3). Improvement in air quality due to measures to control automobile exhaust emissions may contribute to a decrease in the prevalence of asthma, even if many other factors, including advances in prevention and treatment methods, may also be associated. However, the concentrations of TRAP, including NO_2_, remain higher in roadside areas with heavy traffic than in the general environment (Fig. 1b), and these health effects should be considered in the future.

## Health effects of fine PM (PM_2.5_)

### (1) Characteristics of fine PM (PM_2.5_) and air quality standard

Among airborne PM, a mixture of different sized substances, fine PM with an aerodynamic diameter ≤2.5 µm is called PM_2.5_, which comprises primary particles that are directly emitted from motor vehicles and fuel combustion and secondary particles formed through atmospheric chemical reactions involving gaseous precursors [2]. The inhalation of PM_2.5_ which reaches the alveolar regions of the lungs, is a major concern [2, 26]. In Japan, several epidemiological studies were conducted on the health effects of exposure to PM_2.5_ by a study group established by the Japanese Ministry of the Environment from 1999 to 2006, and these reports were released in 2007 [27]. The author conducted some studies on the short-term effects of exposure to PM_2.5_ on respiratory system as a member of the study group [28, 29].

The participants in the first study were children with asthma hospitalized in a suburban city. Peak expiratory flow (PEF) was measured every morning and evening during the study period. Increased 24-h mean concentration of PM_2.5_ was associated with a decrease in both morning and evening PEF, and the changes per 10 µg/m^3^ increments of PM_2.5_ were −3.0 and −4.4 L/min, respectively. The PEF also showed significant associations with lags in hourly concentrations of PM_2.5_, both in the morning and evening [28]. However, primary care visits (PCVs) due to asthma attacks were not associated with the 24-h mean concentration of PM_2.5_, although O_3_ concentrations were significantly associated with PCVs for asthma attacks in children [29].

A cohort study was conducted to evaluate the effects of long-term exposure to PM_2.5_. Respiratory symptoms among 3-year-old children in seven regions of Japan were surveyed every year for five years. The annual average PM_2.5_ concentrations during the follow-up period were not related to either prevalence or incidence of respiratory symptoms among children aged 3–7 years [27]. Another prospective study evaluated the effects of long-term exposure to ambient PM on mortality in six areas. Mortality due to lung cancer was significantly associated with SPM, NO_2_, and SO_2_ concentrations after adjusting for confounding factors including smoking [30].

In Japan, the air quality standards for PM_2.5_, in which the annual and 24-hour averages of PM_2.5_ should be ≤15 and ≤35 µg/m^3^, respectively, were established in 2009 [31], and PM_2.5_ concentrations have been measured continuously at nationwide monitoring stations (Fig. 1d). In January 2013, extremely high concentrations of PM_2.5_ were observed around Beijing, China. During this period, PM_2.5_ concentrations were transiently elevated in western Japan due to transboundary air pollution, exacerbating concerns about their health effects. The association between air pollutants, including PM_2.5_, and PCVs caused by asthma attacks in Himeji City, western Japan, was examined. The PM_2.5_ concentrations from January to March 2013 were slightly higher than those in the corresponding periods in 2011 and 2012, but no association between daily PM_2.5_ concentrations and PCVs due to asthma attacks was observed. In contrast, an increase in 24-h mean O_3_ concentration before PCVs was associated with PCVs due to asthma attacks (OR = 2.31 [95% CI: 1.16, 4.61] for 10-ppb) [32]. Thereafter, the PM_2.5_ concentrations declined in Japan and met the air quality standards for PM_2.5_, almost nationwide [31] (Fig. 1d). The lockdown during the COVID-19 pandemic in 2020 reduced air pollution levels, including those of PM_2.5_, in many countries [33]; however, the change was very small in Japan because the concentrations were already quite low.

Recently, the effects of short- and long-term exposure to PM_2.5_ on human health, such as respiratory, cardiovascular, and nervous systems impacts, have been observed at even lower concentrations than previously reported [26]. Based on these evidences, the WHO updated the air quality guideline level for PM_2.5_ in 2021, in which the recommended annual and 24-hour averages of PM_2.5_ are ≤5 and ≤15 µg/m^3^, respectively [2].

### (2) Health effects of chemical composition of PM_2.5_

As mentioned previously, the concentration of PM_2.5_ has been declining in Japan. However, individuals with respiratory diseases such as asthma are considered more susceptible to air pollutants. Even if the concentrations of PM_2.5_ were considerably lower than the air quality standard level, their short-term effects on pulmonary function and wheezing remained [34–36]. Ambient PM_2.5_ comprises various biological and chemical components, the health effects of which are unclear. To investigate the relationship between the components of PM_2.5_ and their health effects, various components, including endotoxins [37–40], carbons [41], ionic species, and metallic elements [42–45] in PM_2.5_, were analyzed.

A panel study was repeatedly conducted twice a year for one month each in spring and fall from 2014 to 2016 on an isolated island in the Seto Inland Sea, Japan. Daily measurements of PEF and FEV_1_ were performed in 48 healthy college students. PM_2.5_ samples were collected every 24-h from the rooftop of the school building, and the concentrations of mass and 35 chemical components in PM_2.5_ were analyzed [46, 47]. Significant associations were observed between several components of PM_2.5_ and decreased pulmonary function. Among the ionic components, sulfate was strongly related to the decrease in PEF and FEV_1_ (−4.20 L/min [95% CI: −6.40, −2.00] and −0.04 L [95% CI: −0.05, −0.02] for an interquartile range increase, respectively). Among the elemental components, potassium induced the greatest reduction in PEF and FEV_1_ [47]. These results suggest the effects of PM_2.5_ components emitted from numerous ships in the Seto Inland Sea and field burning. Public health measures aimed at reducing the levels of substances identified in these sources may prevent these adverse effects.

### (3) Assessment of the exposure to PM_2.5_

In many previous epidemiological studies, exposure to air pollutants, including PM_2.5_, has been assessed using data from a monitoring station near an individual’s residence. However, the estimation of personal exposure to air pollutants is desirable for a precise evaluation of their effects. Prediction models have been developed to estimate the concentrations of air pollutants at high resolution [48–50].

A large-scale birth cohort study, “the Japan Environmental and Children’s Study (JECS),” has been underway since 2011 to assess the effects of various environmental factors on children’s health and development [51]. The JECS has been conducted in 15 regions of Japan. Hyogo Medical University, to which the author belongs, included approximately 5,000 participants in Amagasaki City, Hyogo Prefecture [52, 53]. The assessment of the effects of exposure to air pollution was one of the objectives of the JECS, and ambient concentrations of key air pollutants were estimated at a 1 × 1 km resolution for application to the participants [54]. Additionally, new prediction models for the daily concentrations of PM_2.5_ mass and major components (sulfate, nitrate, ammonium, EC, and organic carbon) in the Kansai region have been developed [55]. Exposures to PM_2.5_ mass and major components were estimated during pregnancy and early childhood for each participant in Amagasaki City. An increase in the estimated exposure to nitrate during pregnancy was significantly associated with wheezing at 8 years of age (OR = 1.64 [95% CI: 1.10, 2.47] for an interquartile range increase). Estimated exposure to sulfate and ammonium during pregnancy was also significantly associated with allergic sensitization [56]. The components associated with wheezing and sensitization were primarily derived from automobiles and biomass combustion. This study included only participants covered by the Hyogo Regional Center of the JECS, and the difference in exposure levels to PM_2.5_ was small when compared to those across Japan, rendering it insufficient to adequately detect the influence of exposure. The JECS is being implemented in 15 regions, and it is desirable that air pollutant exposure across Japan is evaluated and its effects clarified in the future [51].

Recently, the effects of air pollution on the neurodevelopment of children have attracted increasing attention [57]. Among the children who participated in the JECS, the estimated exposure to PM_2.5_ during pregnancy and early childhood was associated with an increase in externalizing problems at 6 years of age [58]. The effects of pre- and post-natal exposure to TRAP on various outcomes, including allergies, infections, and brain functional network, in children and adolescents have also been reported [59–61]. These results emphasize the importance of addressing air pollution to prevent its adverse effects.

In addition, a randomized crossover intervention study was conducted to evaluate the effects of air purifier use on the indoor environment and respiratory systems of adults. The results revealed that the use of air purification decreased indoor PM_2.5_ and endotoxin concentrations in ordinary homes, but had no demonstrable impact on improving health [62, 63]. The lack of health benefits by improvement in the indoor environment may be due to naturally low concentrations of PM_2.5_ in general Japanese homes.

## Health effects of photochemical oxidants (Ox)

Ox is secondarily produced in the atmosphere through the reaction of volatile organic compounds with NOx in the presence of sunlight, which causes photochemical smog [64]. The concentrations of Ox show a long-term increasing trend owing to the effects of temperature increases and ultraviolet radiation caused by climate change. Ox pollution is not limited to urban districts, but is widespread throughout the world. In Japan, the concentration of Ox exceeds the air quality standard at almost all monitoring stations [31] (Fig. 1e).

O_3_, which is the main constituent of Ox, has a strong oxidizing capacity and adversely affects the respiratory system. It has been previously reported that O_3_ exposure decreases pulmonary function and accelerates bronchial hyperreactivity in mice sensitized to ovalbumin [65, 66]. In human studies in which pulmonary function and airway inflammation markers were repeatedly measured in healthy adults, an increase in the daily O_3_ concentration decreased pulmonary function and aggravated airway inflammation [36, 67, 68].

Additionally, an increase in PCVs due to asthma attacks was associated with increased O_3_ concentrations in studies conducted in Chiba and Hyogo Prefectures [29, 32, 69]. An analysis of 3-year pooled patients in Himeji City, Hyogo showed that PCVs due to asthma attacks were associated with daily O_3_ concentrations before PCVs from April to June (OR = 1.17 [95% CI: 1.01, 1.35] for a 10 ppb increase) and daily PM_2.5_ concentrations from December to March (OR = 1.16 [95% CI: 1.01, 1.33] for a 10 µg/m^3^ increase), suggesting the effects in season when the concentrations of each pollutant were high [70].

The long-term effects of O_3_ on total and respiratory mortality have been also reported [71]. Although the evidence level is considered limited, the WHO established a new air quality guideline level for a long-term peak-season average O_3_ in 2021 [2]. However, the long-term effects of O_3_ on human health require further investigation. It is hoped that these studies will provide scientific evidence for the prevention of adverse effects of O_3_.

## Epidemiological studies using biomarkers

Many epidemiological studies have evaluated the health effects of air pollution using descriptive methods with existing materials such as vital statistics. However, the author has conducted field studies to investigate the effects of air pollution using primary data collected from the population. Field studies have evaluated the health effects of respiratory and allergic diseases using questionnaires and pulmonary function tests. For more sensitive and objective evaluation of the effects of air pollution, the use of biomarkers in biological samples is desirable.

Animal experimental studies have been conducted to develop new biochemical indicators of the adverse effects of exposure to air pollutants such as NO_2_ and O_3_ [65, 66, 72, 73] and attempts have been made to utilize the findings from animal experiments in epidemiological studies. To date, associations between exposure to air pollutants and changes in serum biomarkers such as inflammatory proteins, including high-sensitivity C-reactive protein and several cytokines, have been observed [74–82]. Currently, the focus is on fractional exhaled nitric oxide (FeNO) which is a biomarker of airway inflammation, and an offline method for valid FeNO measurements in field studies has been developed [83]. Short-term effects of exposure to PM_2.5_, O_3_, or both on FeNO levels in healthy adults have been observed [62, 63, 68].

## Conclusions

This article reviews the epidemiological studies on the health effects of air pollution in Japan, focusing on those conducted by the author. After the reduction in air pollution from large-scale factories and power plants during periods of high economic growth, the effects of TRAP on respiratory diseases caused by increased automobile traffic have become a concern. However, the concentrations of TRAP decreased due to the control measures for automobile exhaust. Recently, the effects of PM_2.5_ and O_3_ have been concerning. These air pollutants consist of not only emissions from primary sources but also secondary formations in the atmosphere, which indicates the impact of climate change. Additionally, air pollutants, including PM_2.5_ and O_3_, are known to accelerate global warming. Therefore, air quality control measures and climate change adaptation and mitigation strategies are synergistic, and will have co-benefits on human health [84]. It is hoped that global efforts will be made to protect populations from the health risks caused by these air pollutants.

## Abbreviations

CO: carbon monoxide
CI: confidence interval
EC: elemental carbon
PM_2.5_: fine particulate matter
FEV_1_: forced expiratory volume in 1 s
FeNO: fractional exhaled nitric oxide
JECS: Japan Environmental and Children’s Study
NO_2_: nitrogen dioxide
NOx: nitrogen oxides
OR: odds ratio
O_3_: ozone
PM: particulate matter
PEF: peak expiratory flow
Ox: photochemical oxidants
PCVs: primary care visits
SO_2_: sulfur dioxide
SPM: suspended particulate matter
SORA: Study on respiratory diseases and automobile exhaust
TRAP: traffic-related air pollution
WHO: World Health Organization

## Declarations

### Availability of data and material

No original data was used because this is a review article.
